# Study on visual matching cognition of automobile interior and exterior

**DOI:** 10.1371/journal.pone.0314937

**Published:** 2024-12-11

**Authors:** Yingjie Huang, Bofan Zhang, Jiayu Liu

**Affiliations:** 1 College of Publishing, University of Shanghai for Science and Technology, Shanghai, China; 2 General Motors, Digital Experience Department, Warren, Michigan, United States of America; SRM Institute of Science and Technology (Deemed to be University), INDIA

## Abstract

This work aims to study the visual matching cognition of people with different professional backgrounds for automobile interiors and exteriors, using gasoline-powered automobiles and electric automobiles as the research objects. First, three classes of automobiles, B-class, C-class, and sport-type class automobiles, of different brands, with large differences in styling and styles, were taken as the experimental sample sources, and high-definition interior and exterior pictures of the two power types of automobiles were randomly selected as the experimental samples. The participants were then divided into expert and general user groups according to their professional background. The two groups of participants were asked to conduct a total of 3 groups of visual matching cognition experiments on the interior and exterior of gasoline-powered automobiles, electric automobiles, and automobiles mixed with the same number of gasoline-powered automobiles and electric automobiles. The statistical analysis of the data of the above three groups of experiments revealed that there was no significant difference between the two groups of participants in terms of the visual matching cognition of the interior and exterior of the gasoline-powered automobiles and electric automobiles. When the two types of automobiles were mixed for the experiment, the participants still showed visual matching cognition for the interior and exterior, but there were obvious differences in cognitive abilities between the two groups. Research has shown that both general users and experts have the ability to perform visual matching of automobile interiors and exteriors, however, the cognitive ability of general users is weaker than that of experts. The visual matching cognition of the automobile interior and exterior is not affected by the power type or the automobile type.

## Introduction

In the field of automobile design, automobile styling is an important channel through which automotive brands transmit their brand image to consumers. McCormack et al. [1] studied the styling language and brand relationship of Buick, and reported that unique and easily recognizable automobile styling cognition is conducive to the construction of an automobile brand image. In the field of automobile styling design, designers maintain a company’s brand image and visual recognition by capturing and constructing the explicit and recessive elements defined by automobile aesthetics [2–4]. In automobile products, automobile styling mainly consists of automobile interiors and exteriors. Consistency is an important indicator of product alignment, recognition, and segmentation, including shape matching [5]. Koizumi and colleagues [6] reported that maintaining the color and shape matching of the interior and exterior of an automobile is one of the basic demands of consumers. Therefore, automobile products with matching interior and exterior shapes play an important role in maintaining and building a brand’s image and promoting the major automaker’s products [7–9]. Whether general users can have visual matching cognition of an automobile shape becomes the key to the success for the products launched in the market by automobile manufacturers.

With respect to visual matching cognition in automobile styling, different scholars have carried out research from different angles. Catalano [2] proposed the ontology of automobile styling cognition for automobile product styling, and further summarized the theory. He believed that styling cognition can be divided into two parts: volume cognition in a three-dimensional form, and shape cognition in a two-dimensional form. In addition to the styling form of the automobile interior, the "three-dimensional form" is the interior structure that represents the space volume of the automobile interior. The "two-dimensional form" represents the surface and graphics presented by the styling of the various interior parts. The interior shape of an automobile includes two parts: an automobile interior structure and an automobile interior shape. Notably, the so-called automobile interior structure refers to the visual structure composed of the A, B, and C columns, and the ceiling door components that build the automobile interior space, rather than the structure in the engineering sense. Stylidis et al. studied the perceived quality of automobile exterior lighting (headlights, taillights/signal lights), interior lighting (including trunks) and day lighting outlets (DLOs) [10]. In automobile design, in the fields of color, material, and finishing (CMF) design, the consistency of the CMF and the selection and combination of chromaticity variables hold crucial significance. This is because abstract concepts like emotional attributes need to be expressed, making the richness of performance even more critical [11–13]. Zhao et al. [14] proposed the wholeness and diversity of automobile interior design styles on the basis of their research on the aesthetics and style of the automobile interior. Wholeness is a sense of order in interior design, whereas diversity is composed of interior parts (multipart). The results show that the overall shape of the front face of the automobile, the shape and type of the front intake grill, and the outline of the headlamp are the three most important factors that affect the cognitive results, whereas cultural differences do not have a significant effect. This research shows that local shape cognition is the basic path of overall shape cognition, and that the consistency between the local shape and the overall shape is the condition for the overall shape to be correctly recognized, which is not affected by cultural background [15–18]. Luo et al. [19] adopted Kansei engineering to study the perception matching between automobile wheel hubs and automobile shapes, and the results showed that there was indeed a matching relationship between the two shapes, and that this relationship had a positive promoting effect on the market performance of the product. Yang et al. [20] used a clustering algorithm to study the formal aesthetic consistency of the automobile body and wheel matching, and proposed an "aesthetic matching threshold method". Through a perception experiment, the formal aesthetic consistency result of aesthetic matching, in which the model and wheel hub are more visually coordinated, was obtained. The proposed "aesthetic matching threshold method" can be used to study the matching relationships between complicatedly combined products, and their matching quality, to help designers, businesses, or individuals find suitable solutions [20]. These studies have shown that people can generate a broad sense of visual matching cognition for the styling of automobile products.

In the above research, scholars have focused mainly on the matching between styling elements in the automobile interior or the exterior but have not directly focused on the visual matching cognition between the automobile interior and the exterior. However, a complete automobile product is composed of an automobile interior and exterior. With the development of automobile technology, the complexity and information relevance of the interface of the automobile interaction system are constantly increasing [21–23], and this design trend, to meet the needs of automobile interaction, has led to significant changes in the visual matching cognition of the automobile interior. In particular, in recent years, many electric automobiles have been listed, and the application of new technologies (such as touch control, intelligent surfaces, machine vision, etc.) in human‒automobile interactions by major automakers has made it possible for designers to achieve a better driving experience. Therefore, major automakers have gradually taken the driving experience of the automobile as an important goal of model development. To realize the functions of these driving experiences, the interior and exterior of the automobile have spawned many new styling languages.

This study focuses on different types of gasoline-powered vehicles and electric vehicles, and employs a questionnaire survey method to investigate the visual matching issues related to automobile interior and exterior styling among people from various backgrounds. The study addresses two scientific questions: 1. Does a cognitive disparity exist between general users and experts regarding the visual matching cognition of automobile interior and exterior styling? 2. Is the visual matching cognition between the automobile interior and exterior influenced by the type of powertrain and the vehicle category?

The research value of this article lies in the fact that new energy vehicles, represented by electric vehicles, including artificial intelligence and human‒computer interaction technology, have given rise to a large number of new exterior and interior style designs. With the coexistence of gasoline-powered vehicles and electric vehicles in the current market, research on the visual matching cognition of automobile interior and exterior styling can help companies and designers better grasp the correct design direction and convey their design intentions. Furthermore, effectively meeting the user experience and aesthetic experience of electric vehicles, promoting user acceptance of electric vehicles, and achieving environmentally friendly automotive design concepts, are important.

## Materials and methods

### Visual matching cognitive experiment design for automobile interiors and exteriors

#### Participants

A total of 106 participants participated in the experiment. After review by the Ethics Committee of the University of Shanghai for Science and Technology, it is found that the experiment does not involve the collection of personal health information or privacy of the participants. The experiment does not involve the personal health or privacy collection of the participants. The recruitment of participants for the experiment is based on the principles of voluntary and informed consent. The participants ranged in age from 21 to 55 years, with 57% being male and 43% being female. Among them, individuals with a university degree or above accounted for 75% of the sample. Thirty-one people have backgrounds in automotive design education (hereinafter referred to as the expert group), whereas the remaining 75 people were classified as ordinary users. Sixty-three people in the general user group had driving experience, as follows: 8 had less than 1 year of driving experience, 32–5 years of driving experience, 12–10 years of driving experience, and 11 had more than 10 years of driving experience. To make the experiment more universal, 12 people without a design background or driving experience were randomly invited to participate in the experiment (belonging to the general user group).

#### Sample selection

The types of powertrains used for automobiles can be divided into gasoline-powered vehicles, electric vehicles, and hybrid vehicles [24, 25]. Because most mainstream hybrid models currently available on the market are based on gasoline-powered models with modified power units (such as the Honda Accord, Mercedes Benz E-Class, etc.), the interior design is very similar to that of the gasoline-powered models, with only differences in the design details. Therefore, the scope of this study is limited to gasoline and electric vehicles. Taking five-seating non-SUV automobiles as the benchmark automobile type in the experiments, this category of automobile has a large difference in style, and the highest market share of all the automobile categories, and is more in line with traditional people’s cognition of the shape of automobiles. At present, in the automotive industry, cars are classified into categories A, B, C, D, etc., on the basis of indicators such as vehicle size and power [26, 27]. In terms of the selection of specific experimental materials, the B-class, C-class, and sport-type classes are selected from gasoline-powered automobiles and electric automobiles, respectively. These three classes of automobiles have the widest market share, with the most related products launched by major manufacturers, and significant differences in styling. Because electric automobiles have many new styling languages compared with gasoline-powered automobiles, taking the three classes of the two power types of automobiles as test samples can effectively improve the universality of the test results [28, 29].

First, from the corresponding three classes of gasoline-powered automobiles, a total of 6 test samples composed of two automobiles of each class were randomly selected for Experiment 1. Similar to Experiment 1, from the corresponding three classes of electric automobiles, a total of 6 electric automobiles composed of two automobiles of each class were randomly selected as test samples, which were referred to as Experiment 2. Finally, when gasoline-powered and electric automobiles appear simultaneously in the experiment, in order to further investigate the matching cognitive ability of the two test groups and avoid interference from Experiments 1 and 2, a total of 12 automobiles (different from those in Experiments 1 and 2) were randomly selected from the C-class, B-class, and sport-type gasoline-powered and electric automobiles, respectively, as test samples. The number of automobiles of the two power types was the same in each test. This experiment was called Experiment 3.

**Fig 1:**
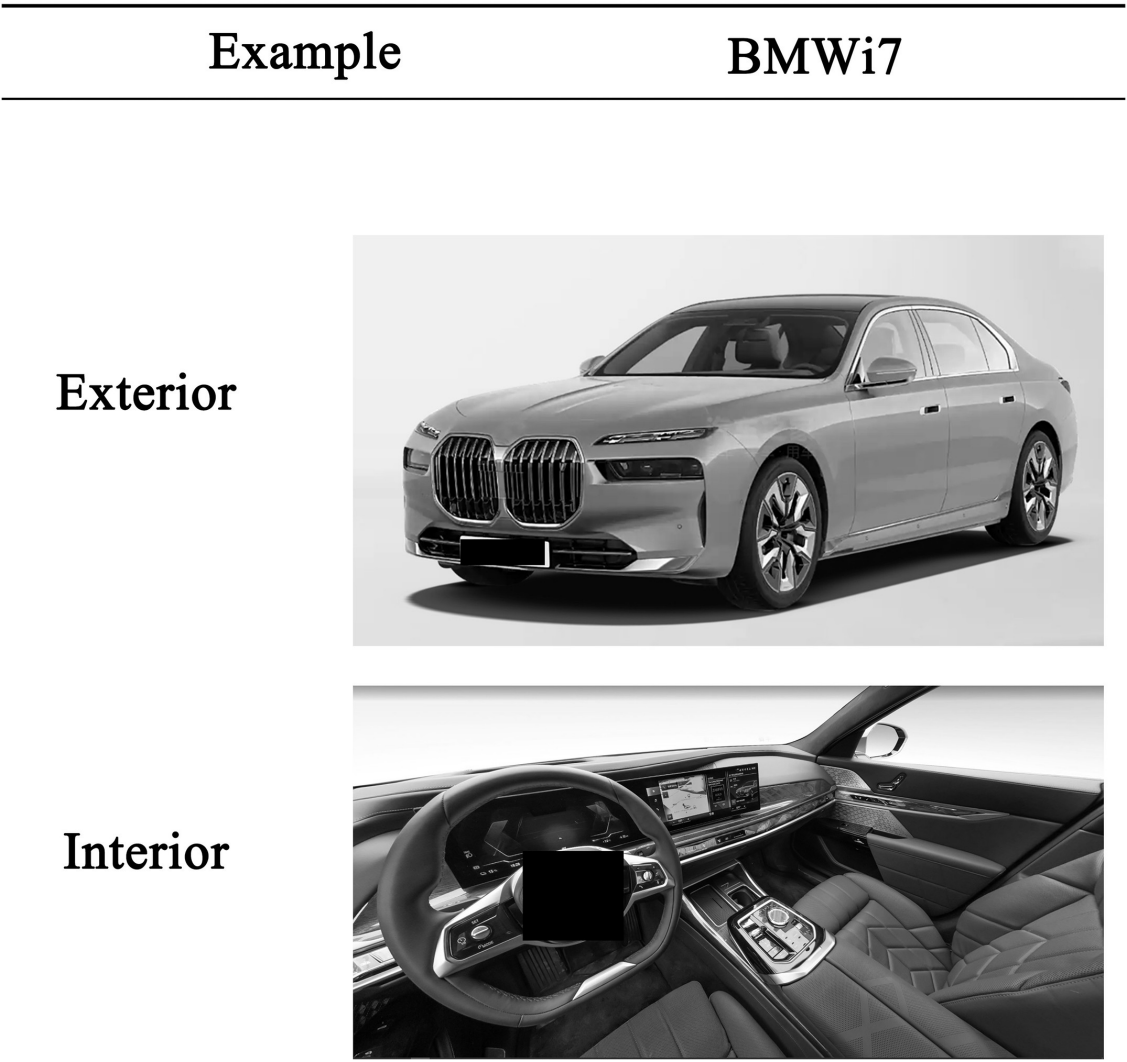
Experimental materials (after color removal).

Generally, the top view angle of the main driving position of the automobile interior, and the first 45-degree view angle of the automobile exterior, contain the most abundant styling information (visual information and volume information) [30, 31]. Therefore, the exterior pictures of the first 45-degree view angle of the automobile, and the high-precision pictures of the top view angle of the main driving position of the automobile interior, were selected as the experimental samples (Fig 1). To avoid visual judgment interference on the styling of the automobile caused by materials and other factors in the experimental sample images, other suggestive factors, such as automobile window glass, steering wheel, and brand logos embedded in the front grille, was excluded from the experimental materials. These logos can reflect the external environment, ensuring that the images do not contain any elements that may affect participants’ judgment. In addition, the color of the experimental materials affects the participants’ understanding of the interior and exterior styling of the automobile [32, 33]. To eliminate the influence of color on the experiment, the experimental samples were subjected to color removal treatment. All the experimental samples (after color removal) used in Experiments 1 and 2, and all the experimental samples used in Experiment 3, are shown in auxiliary S1 and S2 Figs.

### Experimental process

**Fig 2:**
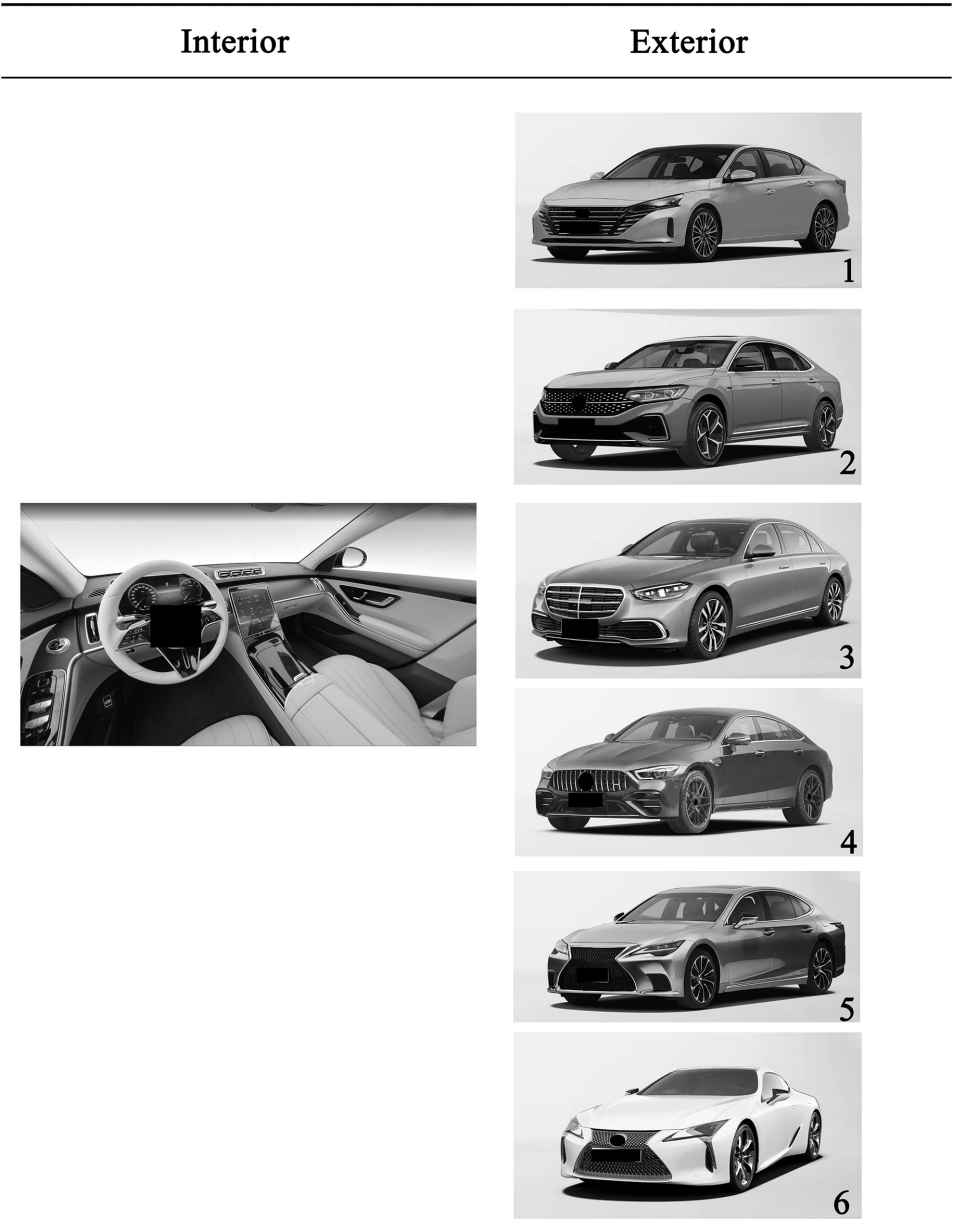
Sample test questions (after color removal, interior: Benz S400L; exterior:1, Nissan Altima; 2, VW Passat; 3, Benz S400L; 4, Benz AMG; 5, Lexus LS500H; 6, Lexus LC 500H).

The experiment was conducted via a questionnaire survey. To improve the accuracy of the experimental results, all the experimental materials were randomly introduced. For Experiment 1 and Experiment 2, a picture of the interior of a certain model is displayed in each question, and the exterior pictures of 6 different automobiles are displayed in the options. Only one choice for each question exactly matches the question stem (the interior and exterior belong to the same class of automobile). For Experiment 3, the question stem shows an automobile interior picture of a certain model, the options show 12 different classes of exterior pictures (6 gasoline-powered automobiles and 6 electric automobiles), and only one option matches the question stem. In the process of the experiment, each question requires the participants, by using their intuition, to choose the option that matches the question stem in the shortest time. The questions were multiple choices, and each participant had to choose at least one, and up to three, choices that they thought matched the question stem. Each question shows a picture of an automobile’s exterior that only matches that choice. In the rules of data statistics, the answer for which the interior completely matches the exterior is called the best answer. For example, the question diagram shown in Fig 2 shows the interior picture of a Mercedes Benz S400L, which is a C-class automobile, and the correct matching exterior is No. 3. The answer for which the same class exterior is selected as the matching class is called the acceptable answer; for example, No. 5 is the exterior of the C-class automobile, the Lexus LS500H. If the participant selects option No. 5, this option is the acceptable answer (in Fig 2, the names of the interior and exterior will not be shown in the formal test). The remaining answer choices were all judged to be wrong answers. This setting can best reflect the true cognitive situation of the test participants, and avoid data distortion and omission due to the limited choice of questions.

## Results and discussion

### Experimental results

A total of 106 questionnaires were sent, and 106 were received. All the questionnaires were true and effective. The expert group submitted 31 questionnaires, and the general user group submitted 75 questionnaires. The experimental results are shown in Tables 1 to 3. Considering that the number of answers for each participant ranges from 1 to 3, including the best answer, acceptable answer, and wrong answer, the correct rate in the table is the sum of the percentages of the best answers and acceptable answers. In addition to providing the percentage of correct answers, the table also provides the percentage of incorrect answers. Notably, for Experiments 1 and 2, there is 1 best answer and 1 acceptable answer for each question, and there are 4 wrong answers to choose from. Therefore, the percentage weight of the correct answers is set to 1, whereas the percentage weight of the wrong answers is set to 0.5. For Experiment 3, there is 1 best answer and 1 acceptable answer for each question, and 10 wrong answers to choose from, so the percentage weight for choosing the right answer in Table 3 is set to 1, whereas the percentage weight for choosing the wrong answer is set to 0.2.

**Table 1:** Results of visual matching cognition Experiment 1 on automobile interior and exterior*.

Question No.	Correct rate (%)	A Data statistics and results	Error rate (%)	Correct rate (%)	B Data statistics and results	Error rate (%)
1	100.00		59.68	94.98		66.83
2	83.85		69.35	86.51		67.59
3	103.18		51.61	88.31		64.62
4	74.19		62.90	83.89		65.80
5	90.32		59.69	82.36		71.03
6	90.32		62.91	66.34		67.18
	*X*_*1*_ = 90.31%		*X*_*2*_ = 61.02%	*X*_*3*_ = 83.73%		*X*_*4*_ = 67.01%
	*S*_*1*_ = 10.59%		*S*_*2*_ = 5.81%	*S*_*3*_ = 9.59%		*S*_4_ = 2.20%
		*F* = 1.82			*F* = 4.36	
		< *F*_0.05,5,5_			< *F*_0.05,5,5_	
		*S*_*1R*_ = 8.54%			*S*_*2R*_ = 6.96%	
		*t*_*1*_ = 5.94			*t*_*2*_ = 4.16	
		> *t*_0.05,10_			> *t*_0.05,10_	
		There are significant differences			There are significant differences	

* A, expert group; B, general user group; *X*_*1*_, *X*_*2*_, are the averages of the correct rate and error rate of the expert group, respectively; *X*_*3*_, *X*_*4*_, are the average correct rate and error rate of the ordinary user group, respectively; *F*, *F* test calculated value; *F*_0.05,5,5_ = 5.05; *S*_1_, *S*_2_, are the standard deviations of the correct rate and error rate of the expert group, respectively; *S*_*3*_, *S*_*4*_, are the standard deviations of the correct rate and error rate of the general user group, respectively; *S*_*1R*_, *S*_*2R*_ are the merged variance values of the expert group and general user group, respectively; *t*_0.05,10_ = 2.23; the correct rate is the sum of the percentages of the best-choice answer and the acceptable answer.

**Table 2:** Results of cognitive Experiment 2 on the visual matching of the automobile interior and exterior*.

Question No.	Correct rate (%)	A Statistics and results	Error rate (%)	Correct rate (%)	B	Error rate (%)
1	103.23		58.06	94.37		57.15
2	87.10		69.36	93.18		52.38
3	112.90		56.45	89.07		42.86
4	106.45		50.00	85.60		50.00
5	80.64		69.34	66.45		63.10
6	100.0		50.00	79.33		58.34
	*X*_*5*_ = 98.39%		*X*_*6*_ = 58.86%	*X*_*7*_ = 84.67%		*X*_*8*_ = 62.30%
	*S*_*5*_ = 12.19%		*S*_*6*_ = 8.56%	*S*_*7*_ = 10.46%		*S*_*8*_ = 3.50%
		*F* = 1.42			*F* = 2.99	
		< *F*_0.05,5,5_			< *F*_0.05,5,5_	
		*S*_*3R*_ = 10.53%			*S*_*4R*_ = 7.80%	
		*t*_*3*_ = 6.5			*t*_*4*_ = 4.97	
		> *t*_0.05,10_			> *t*_0.05,10_	
		There are significant differences			There are significant differences	

* A, expert group; B, general user group; *X*_*5*_, *X*_*6*_, are the averages of the correct rate and error rate of the expert group, respectively; *X*_*7*_, *X*_*8*_, are the average correct rate and error rate of the ordinary user group, respectively; *F*, *F* test calculated value; *F*_0.05,5,5_ = 5.05; *S*_*5*_, *S*_*6*_, are the standard deviations of the correct rate and error rate of the expert group, respectively; *S*_*7*_, *S*_*8*_, are the standard deviations of the correct rate and error rate of the general user group, respectively; *S*_*3R*_, *S*_*4R*_ are the merged variance values of the expert group and general user group, respectively; *t*_0.05,10_ = 2.23; the correct rate is the sum of the percentages of the best choice answer and the acceptable answer.

**Table 3:** Results of cognitive Experiment 3 on the visual matching of the automobile interior and exterior*.

Question No.	Correct rate (%)	A Statistics and results	Error rate (%)	Correct rate (%)	B	Error rate (%)
1	83.87		43.22	79.70		44.76
2	77.39		44.39	73.59		42.81
3	75.12		45.87	71.53		52.08
4	83.86		37.42	61.88		43.62
5	81.00		38.12	70.68		43.05
6	74.19		40.32	63.53		41.32
7	87.10		40.97	60.18		44.61
8	83.86		37.42	67.12		46.06
9	83.86		35.81	70.22		41.32
10	71.18		53.95	60.02		48.32
11	90.32		35.49	62.93		42.10
12	83.86		45.77	56.95		44.04
	*X*_*9*_ = 81.30%		*X*_*10*_ = 41.56%	*X*_*11*_ = 61.41%		*X*_*12*_ = 44.24%
	*S*_*9*_ = 5.71%		*S*_*10*_ = 5.39%	*S*_*11*_ = 8.49%		*S*_*12*_ = 3.12%
		*F* = 1.06			*F* = 2.72	
		<*F*_0.05,11,11_			< *F*_0.05,11,11_	
		*S*_*5R*_ = 5.55%			*S*_*6R*_ = 6.40%	
		*t*_*5*_ = 16.79			*t*_*6*_ = 6.29	
		> *t*_0.05,22_			> *t*_0.05,22_	
		There are significant differences			There are significant differences	

* A, expert group; B, general user group; *X*_*9*_, *X*_*10*_, are the averages of the correct rate and error rate of the expert group, respectively; *X*_*11*_, *X*_*12*_, are the average correct rate and error rate of the ordinary user group, respectively; *F*, *F* test calculated value; *F*_0.05,11,11_ = 2.81; *S*_*9*_, *S*_*10*_, are the standard deviations of the expert group’s correct rate and error rate, respectively; *S*_*11*_, *S*_*12*_, are the standard deviations of the correct rate and error rate of the general user group, respectively; *S*_*5R*_, *S*_*6R*_ are the merged variance values of the expert group and general user group, respectively; *t*_0.05,22_ = 2.074; the correct rate is the sum of the percentages of the best-choice answer and the acceptable answer.

As shown in Tables 1 and 2, for gasoline-powered automobiles and electric automobiles, the average percentages of correct answers selected by the expert group participants accounted for 90.31% and 98.39% of the total number of answers, respectively, whereas the average error rates were 61.02% and 58.86%, respectively. Accordingly, the average answer rates of the general user group were 83.73% and 84.67%, and the average answer error rates were 67.01% and 62.30%, respectively. For Experiment 3, the average percentages of correct answers selected by the expert group and the general user group were 81.3% and 61.41%, respectively, while the error rates were 41.56% and 44.24%, respectively. Tables 1–3 show that the proportion of participants choosing the correct answer is significantly greater than that choosing the wrong answer for both the expert group and the general user group. To study the differences in the cognitive ability of automobile interior and exterior matching, the mathematical statistics are further discussed in the following sections.

### Mathematical statistical analysis of the experimental results

Statistics is the science of collecting, processing, presenting, analyzing, and interpreting the research data obtained into information to assist in making effective decisions, and plays an important role in scientific research [34, 35]. To test whether there are differences in the cognitive ability of visual matching of the automobile interior and exterior between experts and general users, the *t* test method was used to conduct a significant difference analysis on the data in Tables 1 to 3. In accordance with the method described by Zhang et al. [36], the *F* test was first used to determine whether there were significant differences in the evaluation scores of automobile interior and exterior visual matching between the expert group and the general user group. If the *F* value obtained by the calculation is less than the theoretical value (95% confidence level), there is no difference in the evaluation of the accuracy of the score between the two groups; that is, a *t* test can be performed. The data required for the *F* test and *t* test for the experimental data and the results of their calculations are also listed in Tables 1 to 3.

#### Analysis of the results of Experiment 1

According to Table 1, the correct rate of answers was significantly higher than the wrong rate in both the expert group and the general user group. Through a *t* test statistical analysis of the percentage of correct answers and wrong answers, there is a significant difference shown between the choice of the best answer and the choice of the wrong answer. The results show that the two groups of participants have the ability to visually match the interior and exterior of gasoline-powered automobiles.

#### Analysis of the results of Experiment 2

For electric automobiles, according to the *t* test statistical analysis, there is indeed a significant difference in the choice of the best answer and the choice of the wrong answer, indicating that the participants have the cognitive ability to match the interior and exterior of electric automobiles visually. Notably, from the perspective of average answer accuracy alone, 98.39% of the expert group and 84.67% of the general user group answered correctly, which was significantly higher than the corresponding data in Experiment 1 (in Experiment 1, the average answer accuracies of the expert group and the general user group were 90.31% and 83.73%, respectively). Moreover, the error rate of the two groups’ answers in the electric automobile experiment also decreased. The expert group’s average error rate in Experiment 2 was 58.86%, which was lower than the 61.02% reported in Experiment 1. The corresponding data of the general user group were 62.30% and 67.01%, respectively. This shows that, compared with gasoline-powered automobiles, when facing electric automobiles, the two groups of participants produced stronger visual matching cognition of the automobile interior and exterior.

#### Analysis of the results for Experiment 3

According to Table 3, the statistical results show that the participants in both groups have the ability to visually match the automobile interior and exterior. However, there are significant differences between the expert group and the general user group in terms of the correct answer rate.

The data of Experiment 3 indicate that the average answer accuracy rates of the expert group and the general user group are significantly lower than those of Experiment 1 and Experiment 2, which are 81.30% and 61.41%, respectively. Experiment 3 had 12 answers to choose from, but there was still only one best answer and one acceptable answer, which significantly increased the difficulty of choosing, so it is understandable that the correct rate of answers dropped. The results indicate that people’s cognitive ability is related to the difficulty of the task [37], and as the task difficulty increases, the level of cognition decreases [38, 39].

#### Analysis of cognitive differences between different groups in each experiment

Wang Chin-yi studied the differences in the perceptions, understanding, and responsiveness of product design between experts and students [40]. This study indicated that, compared with students, experts have accumulated experience and professional knowledge in many areas of expertise. Therefore, it is worth studying whether there is a difference in the recognition of car interior and exterior styling matches between expert groups and ordinary user groups.

*Analysis of cognitive differences between different groups in the same experiment*. Statistical methods were used to compare cognitive differences between different groups in the same experiment. The correct rate data from Tables 1 to 3 are taken, the steps of the statistical test are followed, the relevant parameters are calculated, and the results are shown in Table 4.

**Table 4:** Statistical analysis of the data from Experiments 1, 2, and 3*.

Parameters	A	EXP 1 Stats	B	A	EXP2 Stats	B	A	EXP 3 Stats	B
*X*	90.31%		83.73%	98.39%		84.67%	81.30%		61.41%
*S*	10.59%		9.59%	12.19%		10.46%	5.71%		8.49%
*F*		1.10			1.17			1.49	
*S*_*R*_		10.10%			11.36%			7.23%	
*t*		1.13			2.09			6.45	

*A, expert group; B, general user group; EXP 1, Experiment 1; EXP 2, Experiment 2; EXP 3, Experiment 3; *X*, average rate of correct answers; *S*, standard deviation; *S*_*R*_, merge variance

According to Table 4, a *t* test can be performed on all 3 sets of experimental data after the *F* test. For Experiment 1 and Experiment 2, *t*_0.05,10_ = 2.23, the calculated *t* values are all less than *t*_0.05,10_, and there is no significant difference. This shows that there is no significant difference between the expert group and the general user group in terms of the visual matching cognition ability of the interior and exterior of gasoline-powered and electric automobiles.

However, for Experiment 3, *t*_0.05,22_ = 1.71, and the calculated *t* value is 6.45, which is greater than *t*_0.05,22_, and there is a significant difference. This shows that there is a significant difference in the accuracy rates of the two groups. This occurred because when the two different types of powered automobiles were present in each question option, the test difficulty faced by the subjects increased compared with that in Experiments 1 and 2. The average percentage of correct answers of the expert group (81.30%) in Experiment 3 was obviously greater than that of the general user group (61.41%), indicating that the professional background of the expert group played an important role in answering the questions. On the other hand, the 12 participants in the general user group had no driving experience, which exacerbates the cognitive gap between the expert group and the general user group. This result is similar to reports in the literature; that is, there are differences in cognitive strategies between experts and novices regarding reasoning in design [37–39].

*Analysis of differences in the cognitive ability of different groups among the three experiments*. The above experimental results show that both groups have the ability to visually match the interior and exterior of an automobile. However, is there still a significant difference in their cognitive ability across the three experiments? To this end, on the basis of the correct answer rate of each experiment, a statistical analysis was conducted to investigate the differences in the cognitive ability of people from different backgrounds across the three experiments.

*Expert group*. The correct answer rates of the expert group in Experiments 1, 2, and 3 were statistically analyzed, and the results are listed in Table 5. The "statistical analysis" in the table refers to the calculated data of the experimental names on the adjacent left and right sides.

**Table 5:** Statistical analysis of data from the expert groups in Experiment 1, Experiment 2, and Experiment 3 *.

Parameters	Experiment 1	Statistical Analysis	Experiment 2	Statistical Analysis	Experiment 3	Statistical Analysis	Experiment 1
*X*	90.31%		98.39%		81.30%		90.31%
*S*	10.59%		12.19%		5.71%		10.59%
*F*		1.15		2.13		1.85	
*S*_*R*_		16.15%		8.68%		7.58%	
*t*		0.87		7.88		4.75	

* *X*, average rate of correct answers; *S*, standard deviation; *S*_*R*_, merge variance

*Between Experiments 1 and 2*. According to Table 5, between Experiment 1 and Experiment 2, *F*_0.05, 5,5_ = 5.05, *F* < *F*_0.05, 5,5_, the *t* test can be performed; *t*_0.05,10_ = 2.23, the calculated *t* value is equal to 0.87, *t* < *t*_0.05,10_, and there is no significant difference. This finding indicates that there is no significant difference in the visual matching cognitive ability on the automobile interior and exterior of the expert group.

*Between Experiments 2 and 3*. The results of Experiments 2 and 3 are as follows, *F*_0.05, 5,11_ = 4.68, *F* < *F*_0.05, 5,11_. A *t*—test can be performed, where *t*_0.05,16_ = 2.12, *t* > *t*_0.05,16_, and there is a significant difference. This result shows that there is a significant difference in the expert group’s degree of cognition of the interior and exterior matching of gasoline-powered automobiles and electric-type automobiles. The reason may be that, although the expert group can match the interior and exterior of the automobile well in Experiments 2 and 3, respectively, in Experiment 3 (the gasoline-powered automobile and electric automobile samples appear at the same time), the number of samples doubles, but the number of correct answers does not increase, which undoubtedly increases the difficulty of the experiment. This is evidenced by the average correct rate in Table 5. The average accuracy rate of the expert group in Experiment 2 is 98.39%, which is significantly higher than the value of 81.30% in Experiment 3.

*Between Experiments 1 and 3*. For Experiments 1 and 3, *F*_0.05, 5, 11_ = 4.68, *F* < *F*_0.05, 5, 11_, a *t* test can be performed; *t*_0.05, 16_ = 2.12, *t* > *t*_0.05, 16_, there is a significant difference. This indicates that the expert group has a significant difference in the cognitive ability of the visual matching of the automobile interior and exterior in Experiments 1 and 3, and the reason is basically the same as that in Experiments 2 and 3.

*General user group*. Table 6 shows the statistical analysis results of the correct answer rates of the general user groups in Experiments 1, 2, and 3.

**Table 6:** Statistical analysis of the data of the ordinary user group in Experiments 1, 2, and 3*.

Parameters	Experiment 1	Statistical analysis	Experiment 2	Statistical Analysis	Experiment 3	Statistical Analysis	Experiment 1
*X*	83.73%		84.67%		61.41%		83.73%
*S*	9.59%		10.46%		8.49%		9.59%
*F*		1.09		1.23		1.23	
*S*_*R*_		10.03%		9.15%		8.85%	
*t*		0.16		10.17		10.09	

* *X*, average rate of correct answers; *S*, standard deviation; *S*_*R*_, merged variance

*Experiment 1 and Experiment 2*. According to Table 6, between Experiment 1 and Experiment 2, *F*_0.05, 5,5_ = 5.05, *F* < *F*_0.05, 5,5_, the *t* test can be performed; *t*_0.05,10_ = 2.23, *t* < t_0.05,10_, and there is no significant difference. This shows that there is no significant difference in the cognition ability of the general user group on the visual matching of the interior and exterior between gasoline-powered automobiles and electric automobiles.

*Experiments 2 and 3*. The results of Experiments 2 and 3 are as follows, *F*_0.05, 5,11_ = 4.68, *F* < *F*_0.05, 5,11_. A *t*—test can be performed, *t*_0.05,16_ = 2.12; and *t* > *t*_0.05,16_, and there is a significant difference. This result shows that there is a significant difference in the cognitive ability of visual matching of the automobile interior and exterior between Experiments 2 and 3. The reason is similar to the results of the expert group in Experiments 2 and 3. As shown in Table 6, the average correct rate of the general user group in Experiment 2 is 84.67%, which is significantly higher than the value of 61.41% in Experiment 3, with a gap of 23.26%. The difference in the average correct rate of the general user group between Experiment 2 and Experiment 3 is very obvious, and is also related to the increase in the difficulty of Experiment 3 compared with that of Experiment 1 and Experiment 2.

*Experiments 1 and 3*. Between Experiment 1 and Experiment 3, where *F*_0.05, 11,5_ = 4.68 and *F* < *F*_0.05, 11,5_, a *t* test can be performed. When *t*_0.05,16_ = 2.12 and *t* > *t*_0.05,16_, there is a significant difference. The results indicate that the cognition ability of the general user group on the visual matching of the automobile interior and exterior is significantly different between Experiments 1 and 3. The reason is similar to that of the general user group in Experiments 2 and 3.

#### Comparison of experimental results

**Fig 3:**
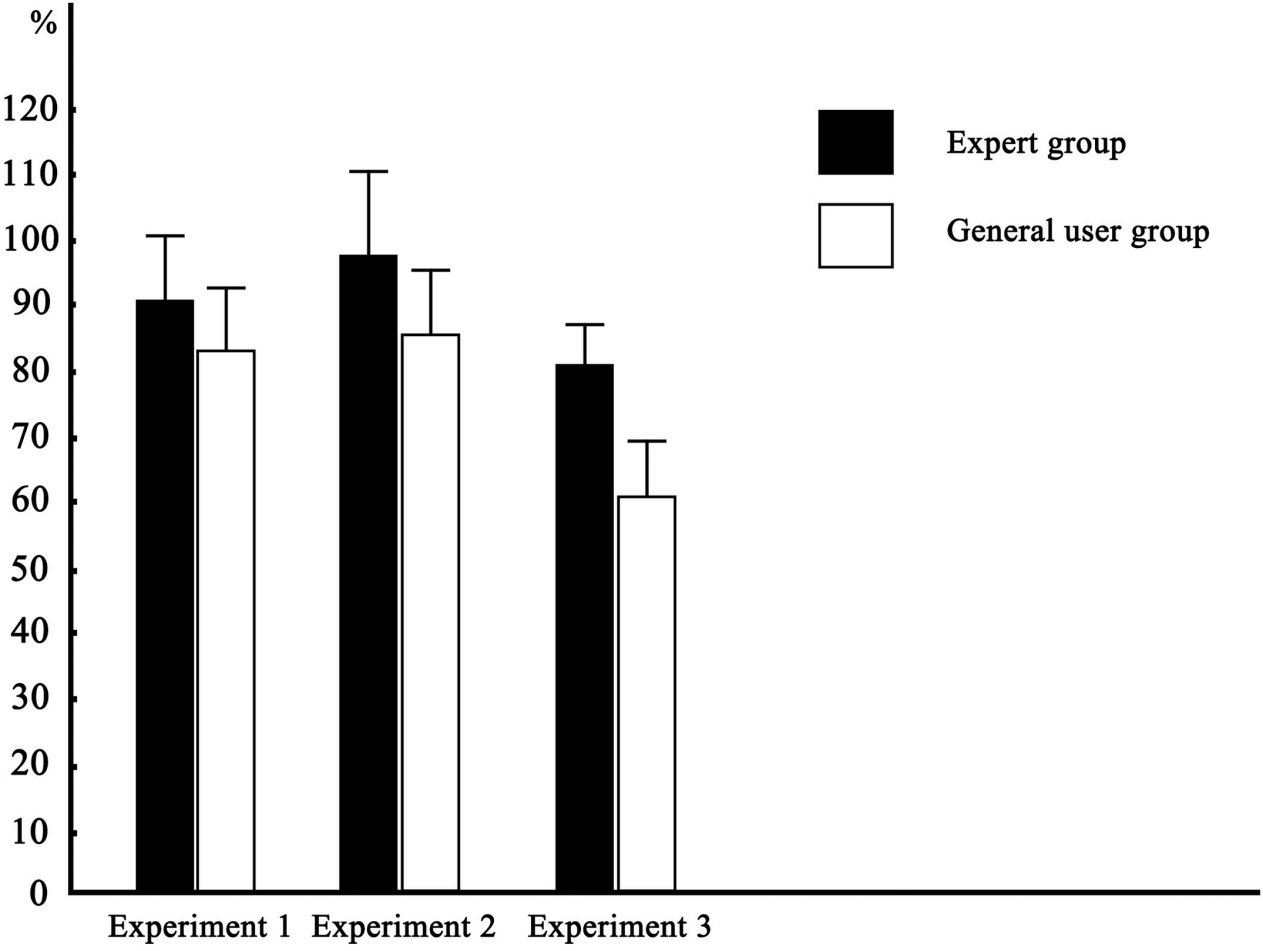
Comparison of the accuracy rates of the answers in the visual matching cognition experiment for the automobile interior and exterior.

Notably, histograms are also good tools for processing experimental data, and can provide a clearer and more intuitive comparison of experimental results [41]. Fig 3 graphically illustrates the average accuracy rates of the expert group and the general user group in Experiments 1, 2, and 3. Fig 3 shows that the accuracy rate of the expert group in each experiment is higher than that of the general user group, which also intuitively shows that professional background (higher sensitivity to styling, greater exposure to automobile models, etc.) has a direct effect on people’s ability to perform visual matching of automobile interiors and exterior styling.

**Fig 4:**
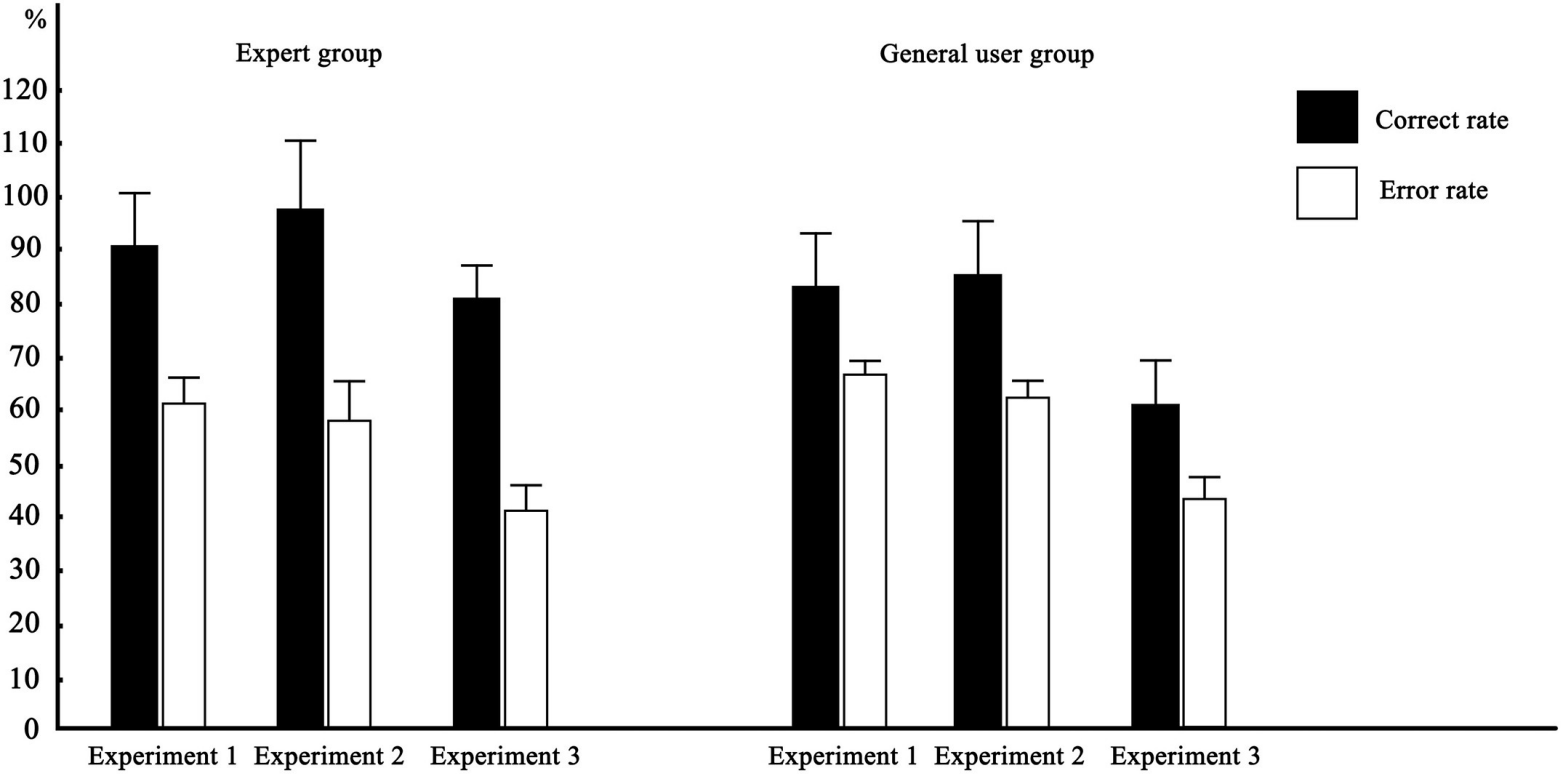
Comparison of the accuracy and error rates of the experiments.

Fig 4 shows the comparison of the accuracy and error rates of the expert group and the general user group in Experiments 1, 2, and 3. As shown in the figure, the correct answer rate is significantly higher than the error rate for both the expert groups and the ordinary user groups. This finding also shows that people have visual matching cognition abilities for automobile interiors and exteriors.

## Conclusions

The test results revealed that the participants in both the expert group and the general user group visually matched the interior and exterior of the automobile, and there was no significant difference between the test results of Experiment 1 (gasoline-powered automobile) and Experiment 2 (electric automobile), which were relatively less difficult. However, in Experiment 3, the expert group and the general user group clearly and significantly differed in terms of the accuracy of the answers, and the expert’s answer accuracy was significantly greater than that of the general users in the experiments. This finding indicates that in a complex cognitive environment, the visual matching cognition ability of the automobile interior and exterior of participants with professional backgrounds is stronger than that of general users. Further analysis of the test results revealed that when the automobile power type was the same, the two groups did not differ in terms of the visual matching cognition ability of the automobile interior and exterior. In Experiment 3, two power types of automobiles were mixed together, and there was a significant difference in the cognitive ability between the two groups, which indicates that, compared with experts with professional backgrounds, the cognitive ability of general users is significantly weakened, and the automobile type does not affect their cognitive ability. The results from Experiments 1 to 3 revealed that visual matching between the interior and exterior of an automobile objectively exists.

In the process of the experiment, the cognitive judgment of the participants for the visual matching of the interior and exterior of the automobile was not based on the exact engineering value but rather on the subjective cognition of the shape. It reflects the consistency of the shape of the interior and exterior of the automobile, and the visual matching process on this basis. Therefore, exploring the basis and mechanism of visual matching when general users and designers recognize the consistency of automobile interior and exterior products, as well as clarifying the reasons leading to the cognitive differences between the two groups, will help designers better convey the design intention to general users in design activities, and will improve the design quality. In addition, similar to styling design, CMF design is also an important part of automotive interior design, and a significant factor affecting people’s cognition of automotive interior and exterior styling. In future research, we will investigate how CMF affects the visual matching cognition of automotive interior and exterior.

## Supporting information

S1 Fig Experiment sample (Experiment1; Experiment2; after color removal). (TIF)

S2 Fig Experiment sample (Experiment 3; after color removal). (TIF)
